# A first-in-human, phase 1, dose-escalation study of dinaciclib, a novel cyclin-dependent kinase inhibitor, administered weekly in subjects with advanced malignancies

**DOI:** 10.1186/1479-5876-11-259

**Published:** 2013-10-16

**Authors:** John J Nemunaitis, Karen A Small, Paul Kirschmeier, Da Zhang, Yali Zhu, Ying-Ming Jou, Paul Statkevich, Siu-Long Yao, Rajat Bannerji

**Affiliations:** 1 Mary Crowley Cancer Research Centers, 1700 Pacific Avenue, Suite 1100, Dallas, TX 75201, USA; 2 Merck & Co., Inc, 1 Merck Drive, Whitehouse Station, NJ 08889, USA; 3 Rutgers Cancer Institute of New Jersey and Robert Wood Johnson Medical School, 195 Little Albany Street, New Brunswick, NJ 08903, USA

**Keywords:** Cyclin-dependent kinase, Dinaciclib, Small-molecule inhibitors, Solid tumors, Cancer therapy

## Abstract

**Background:**

Dinaciclib, a small-molecule, cyclin-dependent kinase inhibitor, inhibits cell cycle progression and proliferation in various tumor cell lines in vitro. We conducted an open-label, dose-escalation study to determine the safety, tolerability, and bioactivity of dinaciclib in adults with advanced malignancies.

**Methods:**

Dinaciclib was administered starting at a dose of 0.33 mg/m^2^, as a 2-hour intravenous infusion once weekly for 3 weeks (on days 1, 8, and 15 of a 28-day cycle), to determine the maximum administered dose (MAD), dose-limiting toxicities (DLTs), recommended phase 2 dose (RP2D), and safety and tolerability. Pharmacodynamics of dinaciclib were assessed using an ex vivo phytohemagglutinin lymphocyte stimulation assay and immunohistochemistry staining for retinoblastoma protein phosphorylation in skin biopsies. Evidence of antitumor activity was assessed by sequential computed tomography imaging after every 2 treatment cycles.

**Results:**

Forty-eight subjects with solid tumors were treated. The MAD was found to be 14 mg/m^2^ and the RP2D was determined to be 12 mg/m^2^; DLTs at the MAD included orthostatic hypotension and elevated uric acid. Forty-seven (98%) subjects reported adverse events (AEs) across all dose levels; the most common AEs were nausea, anemia, decreased appetite, and fatigue. Dinaciclib administered at the RP2D significantly inhibited lymphocyte proliferation, demonstrating a pharmacodynamic effect. Ten subjects treated at a variety of doses achieved prolonged stable disease for at least 4 treatment cycles.

**Conclusions:**

Dinaciclib administered every week for 3 weeks (on days 1, 8, and 15 of a 28-day cycle) was generally safe and well tolerated. Initial bioactivity and observed disease stabilization support further evaluation of dinaciclib as a treatment option for patients with advanced solid malignancies.

**Trial registration:**

ClinicalTrials.gov # NCT00871663

## Background

Cyclin-dependent kinases (CDKs) are serine/threonine kinases that regulate progression through the cell cycle [1]. They exist in heterodimeric complexes with cyclins and are activated at different stages of the cell cycle by various cyclins. Eleven CDKs have been identified with distinct functions in controlling the activation of the cell cycle and progression from the G1 phase through mitosis [2]. Phosphorylation of the retinoblastoma (Rb) family of proteins is an important mechanism by which the CDKs regulate cell cycle progression [3]. In addition to their role in cell cycle progression, CDKs also play an important role in transcriptional regulation by phosphorylating the carboxy-terminal domain of the large subunit of ribonucleic acid polymerase II; CDK7/cyclin H and CDK9/cyclin T have been shown to play important roles in transcription initiation and elongation, respectively [4].

Dysregulation of the cell cycle plays an important role in malignant transformation and the development of resistance to chemotherapy [4]. Overexpression or underexpression of the cyclins and CDKs that regulate the cell cycle has been observed in a variety of tumors and proliferative diseases, including melanoma [5], multiple myeloma [6], pituitary adenomas and carcinomas [7], chronic lymphocytic leukemia (CLL) [8], and other solid malignancies [9,10]. This has spurred interest in the development of novel anticancer agents that target CDKs. As anticancer treatments, CDK inhibitors have been found not only to block cell cycle progression but also to promote apoptosis, which leads to cell death. In particular, CDK inhibitors have shown high activity in cell lines from nonproliferative cancers such as CLL and multiple myeloma due to their ability to induce apoptosis [11].

Dinaciclib (MK-7965, formerly SCH727965) is a novel, potent, small-molecule inhibitor of CDK1, CDK2, CDK5, and CDK9 with half maximal inhibitory concentration (IC_50_) values in the 1 nM to 4 nM range, and inhibits CDK4, CDK6, and CDK7 at IC_50_ values in the 60 nM to 100 nM range [12,13]. Dinaciclib was initially selected from a compound screen in a mouse xenograft model, using flavopiridol as the reference [12]. The maximum tolerated dose, defined as the dose associated with 20% weight loss, was 60 mg/kg for dinaciclib versus <10 mg/kg for flavopiridol following once-daily administration for 7 days in nude mice. The dinaciclib minimum effective dose, defined as >50% tumor growth inhibition, was 5 mg/kg versus 10 mg/kg for flavopiridol, yielding a screening therapeutic index of >10 for dinaciclib and <1 for flavopiridol. Although not formally investigated, the strong selectivity for CDKs—but not the closely related serine/threonine kinases—suggests that dinaciclib may target an activated CDK conformation not present in serine/threonine kinases. In vitro, dinaciclib has been shown to suppress phosphorylation of the Rb tumor suppressor protein, to induce activation of caspase and apoptosis, and to inhibit cell cycle progression and proliferation in various tumor cell lines [5,12,14]. Promising antitumor activity following treatment with dinaciclib has also been demonstrated using in vivo mouse xenograft models, with minimal toxic effects at active dose levels [5,12,14,15], and tissue fragments of patient-derived xenografts grown in mice [5,12,14,15].

We conducted a phase 1 study with dinaciclib, administered as a 2-hour intravenous (IV) infusion once every week for 3 weeks followed by a 1-week recovery (28-day cycle), in subjects with advanced malignancies. The primary objectives of this study were to determine the safety, tolerability, maximum administered dose (MAD), dose-limiting toxicity (DLT), and recommended phase 2 dose (RP2D) of dinaciclib, and to assess pharmacodynamic (PD) effects using an ex vivo lymphocyte stimulation assay, Rb protein phosphorylation, and ^18^ F-fluorodeoxyglucose positron emission tomography/computed tomography (FDG-PET/CT).

## Methods

### Study population

This was a nonrandomized, open-label, phase 1 trial (ClinicalTrials.gov identifier #NCT00871663; http://clinicaltrials.gov/ct2/show/NCT00871663; Protocol 04629) of adult subjects (≥18 years) with histologically proven solid tumors, non-Hodgkin’s lymphoma, or multiple myeloma refractory to standard therapy or for which there is no standard therapy. Subjects had Eastern Cooperative Oncology Group (ECOG) performance statuses of 0, 1 or 2 and had to have adequate organ function and laboratory parameters. Subjects were excluded from the study if they had symptomatic brain metastases or primary central nervous system malignancy. Subjects must not have received any radiation therapy within 4 weeks prior to the start of treatment with dinaciclib, or have had a history of radiation therapy to greater than 25% of the total bone marrow. In addition, subjects could not have received previous treatment with an investigational drug or biologic or hormonal therapy within 4 weeks of study treatment; mitomycin, nitrosourea, nilutamide, or bicalutamide within 6 weeks of study treatment; or cytochrome P450 3A4 inhibitors or inducers within 1 week of study treatment. Known human immunodeficiency virus (HIV) and HIV-related malignancy were also exclusion criteria.

The study was conducted in accordance with good clinical practice and the Declaration of Helsinki concerning written informed consent and the protection of rights of human subjects. Before study initiation, the clinical study protocol, any amendments, and the written informed consent forms were reviewed and approved by an independent review board at each study site. Each subject had to provide written informed consent before undergoing any study-related activities.

### Study endpoints and treatment plan

The primary endpoints of the study were to determine the safety, tolerability, MAD, DLT, and the RP2D of dinaciclib, and to assess the PD effects of dinaciclib on peripheral blood lymphocytes. Secondary endpoints included determining the pharmacokinetic (PK) profile of dinaciclib following a single dose and following the third weekly dose, assessment of Rb protein phosphorylation in subject skin biopsy samples, preliminary evaluation of the antitumor activity of dinaciclib, and assessment of tumor metabolic changes in response to dinaciclib treatment via use of FDG-PET/CT.

Dinaciclib was administered as a 2-hour IV infusion on days 1, 8, and 15 of a 28-day cycle. The 2-hour duration of IV infusion was selected based on previous nonclinical toxicity/toxicokinetic studies conducted in dogs that demonstrated acute toxicity following IV push. Subjects continued on treatment until there was disease progression, unacceptable toxicity, or the subject withdrew consent. The trial employed an accelerated titration design [16] starting at a dose of 0.33 mg/m^2^ (one-sixth of the highest nontoxic dose in dogs). Routine antiemetic prophylaxis was administered to patients receiving a dose of 7.11 mg/m^2^ and above, due to nausea and vomiting observed at lower dose levels. Antiemetic prophylaxis consisted of a serotonin-receptor antagonist (eg, ondansetron), with or without dexamethasone, administered prior to treatment with dinaciclib, and modifications were permitted as clinically indicated.

### Toxicity, safety, and tolerability assessments

To determine the MAD of dinaciclib administered as a 2-hour IV infusion, an accelerated titration design was used, whereby at least one subject was treated at each dose level starting with 0.33 mg/m^2^; the dose was doubled in sequential subjects until a DLT was observed or a subject experienced grade 2 toxicity [16]. In the case of an observed grade 2 toxicity, a second subject was enrolled at the same dose level. If the second subject also experienced a grade 2 toxicity, 2 additional subjects were accrued at that dose level for a total of 4 subjects. In the case of an observed DLT, additional subjects were added to the cohort until either a second subject experienced a DLT or 6 subjects were treated at that dose level. If 2 or more subjects experienced a DLT at a given dose, then 3 additional subjects were treated at the previous lower dose, unless 6 subjects had already been treated at that dose. Dose escalations beyond the 1.32-mg/m^2^ dose level were administered in increments of 40% in cohorts of 3 subjects. Each subject was allocated to only a single dose level of drug. Dose delay or modification was permitted based on laboratory and clinical assessment performed on the day of treatment. The RP2D was defined as the highest dose studied, without growth factor support, for which the incidence of DLT was less than 33% (ie, the dose level immediately lower than the MAD), determined based on myeloma and NSCLC mouse xenograft models, which showed complete tumor regression at a dose 33% of the MAD.

Dose-limiting toxicities were determined during the first cycle for each dose level. A DLT was defined as any grade 3 or 4 hematologic toxicity lasting for at least 1 week, or as any grade 3 or 4 nonhematologic toxicity. Untreated nausea and vomiting, fatigue, anorexia, anemia, alopecia, or local reactions were not included in the determination of DLTs and did not alter the escalation schedule, unless inclusion was deemed necessary by the investigator and sponsor. Normal alkaline phosphatase level (< grade 1) at screening that rose to greater than or equal to grade 3; grade 1 or 2 alkaline phosphatase level at screening that rose to grade 4; grade 1 or 2 aspartate aminotransferase (AST) and/or alanine aminotransferase (ALT) levels at screening that doubled from baseline to become greater than or equal to grade 3; and any other abnormal nonhematology laboratory value greater than or equal to grade 3 that required medical intervention to treat, led to hospitalization, or persisted for at least 1 week were also considered DLTs.

Safety and tolerability of dinaciclib were assessed based on review of laboratory test results, electrocardiograms, vital signs, physical examinations, and reported adverse events (AEs). Any abnormal laboratory results that led to hospitalization, resulted in a change in dosing, or were medically significant were reported as AEs. Adverse events were graded based on the National Cancer Institute Common Terminology Criteria for Adverse Events (NCI CTCAE version 3.0) and were coded using the Medical Dictionary for Regulatory Activities (MedDRA version 13.0).

### Pharmacodynamic and pharmacokinetic assessments

The antiproliferative activity of dinaciclib was assessed ex vivo using whole blood samples obtained on days 1 and 15 of cycle 1 (predose and at 2, 3, 4, 6, and 8 hours after the start of infusion), predose on day 8 of cycle 1, and on day 22 of cycle 1. Whole blood isolated from subjects was treated with phytohemagglutinin (PHA) to stimulate cell division in lymphocytes. Following a brief 30-minute exposure to bromodeoxyuridine (BrdU), cells were harvested and stained using an FITC-conjugated antibody specific for BrdU, counterstained with propidium iodide/RNase A, and analyzed using a FACSCalibur flow cytometer. Approximately 35% to 40% of the CD45-positive cells in the whole blood incorporate BrdU following PHA stimulation under conditions defined in this assay, signifying DNA synthesis and cell division. Any subject with less than 5% BrdU incorporation post treatment was classified as a responder to dinaciclib treatment. To explore the relationship between exposure and bioactivity of dinaciclib,%BrdU incorporation was correlated with the amount of dinaciclib found in plasma samples taken at the same time.

Skin punch biopsies (4 mm) were obtained before and 4 hours after treatment with dinaciclib, and fixed in 10% buffered formalin for immunohistochemistry (IHC) analysis. The phosphorylation status of the Rb protein in the proliferative layer of skin and in the proliferating cells at the base of hair follicles was determined by IHC utilizing a rabbit anti-phospho-Rb antibody diluted to 0.83 μg/mL or 0.67 μg/mL. Immunohistochemistry staining was scored 0 (no staining relative to background), 1+ (weak staining), 2+ (moderate staining), or 3+ (strong staining); H-score was calculated as a measure of overall immunoreactivity in a given sample, using the following formula: H-score = (% of cells with 3+) × 3 + (% of cells with 2+) × 2 + (% of cells with 1+) × 1. Subjects were to be categorized as responders if no degree of staining was detected with the anti-phospho-Rb antibody.

Pharmacodynamic effects were further assessed by monitoring decreased metabolic activity following IV infusion of dinaciclib using FDG-PET/CT scans, conducted within 14 days prior to the first dose of dinaciclib and on day 22 of cycle 1, unless treatment was delayed. Metabolic activity data were obtained for research use only and were not used for clinical management of subjects. A 30% reduction in posttreatment standardized uptake value (SUV_max_), in up to 6 lesions prospectively identified at the start of treatment as the most representative metabolically active sites of disease, was used to determine responders and nonresponders to dinaciclib treatment.

Dinaciclib plasma concentrations were analyzed on days 1 and 15 of cycle 1 prior to the start of infusion, and at 1 hour, 2 hours, 2 hours 15 minutes, 2 hours 30 minutes, 3 hours, 3 hours 30 minutes, 4 hours, 5 hours, 6 hours, and 8 hours after the start of the infusion. Additional blood samples for PK analysis were obtained on days 2 and 16 of cycle 1 (between 24 and 28 hours after the start of the infusion), on day 8 of cycle 1, and on day 1 of cycle 2, prior to and 2 hours after the start of the infusion. Plasma concentrations of dinaciclib were determined, as previously described, using validated high performance liquid chromatographic-tandem mass spectrometry (LC-MS/MS) methods [17]. Briefly, plasma samples were fortified with an internal standard (^13^C3)-dinaciclib in 1:1 ratio, loaded into a Water Oasis MCX Solid Phase Extraction plate, washed with phosphoric acid/methanol, and eluted with methanol/ammonium hydroxide. The eluent was evaporated and the extract injected into a LC-MS/MS. The retention time for dinaciclib and the internal standard was 2.5 minutes and detection was performed using a Sciex API 5000 triple quadrupole LC-MS/MS system with a turbo ion spray source. Key pharmacokinetic parameters evaluated for dinaciclib included maximum observed plasma concentration (C_max_), time of maximum plasma concentration (T_max_), area under the plasma concentration-time curve from time zero to infinity (AUC_(I)_), terminal phase half-life (t_½_), clearance (CL), volume of distribution (V_d_), and accumulation ratio (R).

### Tumor response assessment

Antitumor activity of dinaciclib on solid tumors was evaluated using CT or magnetic resonance imaging (MRI) scans and Response Evaluation Criteria In Solid Tumors (RECIST) guidelines [18]. Computed tomography or MRI scans were obtained within 4 weeks prior to the start of treatment with dinaciclib, and were repeated after every 2 cycles and at the poststudy assessment performed 4 weeks after the start of the last cycle.

### Statistical analyses

Demographic and baseline variables for each subject (including primary diagnosis) were tabulated and summarized using descriptive statistics. No inferential analysis of safety data was planned; subjects reporting any AEs, the occurrence of specific AEs, and discontinuation due to AEs were summarized using descriptive statistics. For%BrdU incorporation (PD primary endpoint), the response rate and its 95% 2-sided exact confidence interval (CI) were calculated if 6 or more responders were observed among 10 subjects; a level at which the lower limit of the 2-sided 95% exact CI was expected to be greater than 25%, allowing inference with high confidence that the metabolic inhibition rate was more than 25%. For each dose level, treatment effect on inhibition of lymphocyte proliferation was evaluated by comparing the pretreatment with the posttreatment%BrdU incorporation on days 1 and 15 at specified posttreatment time points (2, 3, 4, 6, and 8 hours after the start of the infusion) using a paired *t*-test (in dose levels with sample sizes ≥3). For secondary endpoints, subjects were classified as responders or nonresponders and the response rate and its 95% CI were determined. Summary statistics (means, standard deviations, coefficients of variation) were calculated using noncompartmental methods with the WinNonlin software (V5.2, Pharsight, NC) for the concentration-versus-time data at each sampling time and for derived PK parameters.

## Results and discussion

### Subject disposition and baseline characteristics

The study enrolled 52 subjects with histologically proven solid tumors for whom there was no known standard therapy or who had disease refractory to standard therapy. Treatment was administered to 48 subjects; 3 subjects were enrolled but did not meet protocol eligibility criteria and were never treated, and one subject who was enrolled did not receive any treatment because of an AE. However, when screening data from these subjects were available for a given measurement, these subjects were included in the corresponding analysis. According to the trial design, all subjects continued treatment until disease progression or treatment discontinuation due to toxicity or at the subject’s request; most trial discontinuations were due to disease progression (28 [58%] subjects) and symptomatic deterioration (8 [17%] subjects).

Table 1 summarizes subject demographics and baseline disease characteristics. The majority of patients enrolled in the study were white (33 [69%] subjects), male (27 [56%] subjects), and younger than 65 years old (30 [63%] subjects), with a mean age of 61.6 years. Most subjects had colorectal cancer (15), followed by non–small cell lung cancer (NSCLC, 5), ovarian cancer (4), breast cancer (3), and melanoma (3). The study population had received a median of 3 (range 0–16) chemotherapy regimens prior to enrolling into the trial.

**Table 1:** Baseline demographic and disease characteristics

**Demographic or disease characteristic**	**Number of subjects (N = 48)**
Sex, n (%)	
Female	21 (44)
Male	27 (56)
Race, n (%)	
White	33 (69)
Nonwhite	15 (31)
Asian	1 (2)
Black or African American	13 (27)
Multiracial	1 (2)
Age, years	
Mean (standard deviation)	61.6 (11.2)
Median	62
Range	39–81
Age, n (%)	
18–64 years	30 (63)
≥65 years	18 (38)
ECOG performance status, n (%)	
0	12 (25)
1	31 (65)
2	5 (10)
Prior chemotherapy regimens, n (%)	
≤2	16 (33)
3–5	23 (48)
≥6	9 (19)
Cancer type, n	
Colorectal	15
NSCLC	5
Ovarian	4
Breast, melanoma	3 each
Prostate, cholangiocarcinoma, adenoid cystic, sarcoma, pancreatic, neuroendocrine	2 each
Esophageal, gastric, gastrointestinal stromal tumor, hepatocellular, pseudomyxoma peritonei, vulvar	1 each

### Toxicity, safety, and tolerability of dinaciclib

A total of 11 subjects were administered doses of dinaciclib ranging from 0.33 to 2.59 mg/m^2^; there were 2 instances of grade 2 toxicity at 1.32 mg/m^2^, but no DLTs were experienced at any of these dose levels. Therefore, subsequent doses were escalated in 40% increments from 1.85 mg/m^2^ up to the MAD that was reached at a dinaciclib dose of 14 mg/m^2^. Two subjects among the 5 treated at the MAD experienced a DLT, one with orthostatic hypotension and one with elevated uric acid (Table 2). A lower dose of 12 mg/m^2^ was tested and was determined to be the RP2D for dinaciclib administered as a 2-hour IV infusion once a week for 3 weeks followed by a 1-week recovery period. A total of 11 subjects were tested at the RP2D dose; one subject experienced septic shock as a DLT. Additional DLTs experienced with dinaciclib included hypokalemia, hypocalcemia, and hypophosphatemia experienced by 1 of 8 subjects treated at the 3.63 mg/m^2^ dose level, and deep vein thrombosis in 1 of 7 subjects treated at the 7.11 mg/m^2^ dose level.

**Table 2:** Administered dinaciclib dose levels and DLTs

**Dose level (mg/m**^**2**^**)**	**Number of subjects**	**Subjects with DLT in cycle 1**	**DLT**
0.33	1	0	None
0.66	1	0	None
1.32	1	0	None
1.85	4	0	None
2.59	4	0	None
3.63	8	1	Hypokalemia, hypocalcemia, hypophosphatemia
5.08	3	0	None
7.11	7	1	Deep vein thrombosis
10	3	0	None
12	11	1	Septic shock
14	5	2	Orthostatic hypotension (1), elevated uric acid (1)

A total of 47 (98%) subjects reported treatment-emergent adverse events (TEAEs; Table 3), and 35 (73%) subjects experienced AEs possibly related to study drug. The most frequently reported treatment-related AEs were nausea (16 [33%] subjects), anemia (10 [21%] subjects), neutropenia (8 [17%] subjects), vomiting (8 [17%] subjects), and fatigue (7 [15%] subjects). At the RP2D (12 mg/m^2^), the most common treatment-related AEs reported by at least 3 (>25%) of the 11 subjects treated at this dose level were anemia (5 [45%]), neutropenia (4 [36%]), fatigue (4 [36%]), nausea (3 [27%]), vomiting (3 [27%]), asthenia (3 [27%]), hyperuricemia (3 [27%]), and pyrexia (3 [27%]). Sixteen (33%) subjects experienced grade 3 or 4 treatment-related AEs, with neutropenia (5 [10%] subjects) and hyperuricemia (3 [6%] subjects) being the most common. Serious AEs (SAEs) were reported in 17 (33%) subjects; the most common SAEs were deep vein thrombosis, sepsis, and anemia, each occurring in 3 (6%) subjects. Not all SAEs qualified as DLTs. No discernible trend regarding tumor type and toxicity was identified (Additional file 1: Table S1).

**Table 3:** TEAE’s occurring in ≥10% of subjects

**Adverse event, n (%)**	**Dinaciclib dose level (mg/m**^**2**^**)**											
	**All doses (N = 48)**		**≤ 5.08 (n = 22)**		**7.11 (n = 7)**		**10 (n = 3)**		**12 (n = 11)**		**14 (n = 5)**	
	**All grades**	**Grade 3–4**	**All grades**	**Grade 3–4**	**All grades**	**Grade 3–4**	**All grades**	**Grade 3–4**	**All grades**	**Grade 3–4**	**All grades**	**Grade 3–4**
Subjects with any AE	47 (98)	29 (60)	21 (95)	11 (50)	7 (100)	4 (57)	3 (100)	1 (33)	11 (100)	8 (73)	5 (100)	5 (100)
Anemia	19 (40)	5 (10)	8 (36)	1 (5)	3 (43)	2 (29)	0	0	6 (55)	1 (9)	2 (40)	1 (20)
Nausea	19 (40)	1 (2)	6 (27)	0	5 (71)	0	1 (33)	0	3 (27)	0	4 (80)	1 (20)
Fatigue	14 (29)	2 (4)	7 (32)	2 (9)	3 (43)	0	0	0	4 (36)	0	0	0
Decreased appetite	14 (29)	0	9 (41)	0	2 (29)	0	0	0	2 (18)	0	1 (20)	0
Urinary tract infection	12 (25)	0	5 (23)	0	2 (29)	0	1 (33)	0	4 (36)	0	0	0
Asthenia	11 (23)	3 (6)	4 (18)	2 (9)	2 (29)	0	0	0	4 (36)	0	1 (20)	1 (20)
Back pain	11 (23)	3 (6)	8 (36)	3 (14)	1 (14)	0	0	0	1 (9)	0	1 (20)	0
Vomiting	11 (23)	2 (4)	2 (9)	1 (5)	2 (29)	1 (14)	2 (67)	0	3 (27)	0	2 (40)	1 (20)
Constipation	11 (23)	0	5 (23)	0	1 (14)	0	2 (67)	0	2 (18)	0	1 (20)	0
Hyperbilirubinemia	9 (19)	5 (10)	5 (23)	3 (14)	0	0	0	0	3 (27)	1(9)	2 (40)	1 (20)
Dehydration	9 (19)	0	8 (36)	0	1 (14)	0	0	0	0	0	3 (60)	0
Neutropenia	8 (17)	5 (10)	0	0	0	0	1 (33)	1 (33)	4 (36)	2 (18)	3 (60)	2 (40)
Abdominal pain	8 (17)	2 (4)	4 (18)	1 (5)	3 (43)	1 (14)	1 (33)	0	0	0	0	0
Increased AST	8 (17)	2 (4)	3 (14)	1 (5)	0	0	0	0	4 (36)	1 (9)	1 (20)	0
Increased ALP	8 (17)	2 (4)	3 (14)	1 (5)	0	0	0	0	4 (36)	0	1 (20)	1 (20)
Pyrexia	8 (17)	0	3 (14)	0	1 (14)	0	0	0	4 (36)	0	0	0
Hyperglycemia	7 (15)	4 (8)	1 (5)	0	0	0	1 (33)	1 (33)	4 (36)	2 (18)	1 (20)	1 (20)
Dyspnea	7 (15)	3 (6)	3 (14)	1 (5)	3 (43)	1 (14)	0	0	0	0	1 (20)	1 (20)
Peripheral edema	7 (15)	0	3 (14)	0	1 (14)	0	0	0	3 (27)	0	0	0
Hypophosphatemia	6 (13)	5 (10)	4 (18)	3 (14)	0	0	0	0	2 (18)	2 (18)	0	0
Hypokalemia	6 (13)	2 (4)	2 (9)	1 (5)	1 (14)	0	1 (33)	0	2 (18)	1 (9)	0	0
Increased ALT	6 (13)	1 (9)	3 (14)	1 (5)	0	0	0	0	3 (27)	0	0	0
Diarrhea	6 (13)	0	3 (14)	0	1 (14)	0	1 (33)	0	1 (9)	0	0	0
Flatulence	6 (13)	0	3 (14)	0	2 (29)	0	0	0	1 (9)	0	0	0
Hyperuricemia	5 (10)	4 (8)	1 (5)	1 (5)	0	0	0	0	3 (27)	2 (18)	1 (20)	1 (20)
Dizziness	5 (10)	1(9)	0	0	3 (43)	0	0	0	1 (9)	0	1 (20)	1 (20)
Abdominal distension	5 (10)	0	3 (14)	0	1 (14)	0	0	0	1 (9)	0	0	0
Weight decreased	5 (10)	0	4 (18)	0	1 (14)	0	0	0	0	0	0	0
Cough	5 (10)	0	3 (14)	0	0	0	0	0	2 (18)	0	0	0

ALP = alkaline phosphatase.

Adverse events are listed by dose level; all grades and grade 3 or grade 4.

Eleven (21%) of the 52 subjects enrolled died during this study. The most common reason for death was disease progression (7 [13%] subjects) considered to be unlikely related to study treatment. Deaths due to AEs occurred in 4 (8%) subjects: one subject assigned to the 7.11-mg/m^2^ dose was never treated and died due to aspiration; one subject who received the 7.11-mg/m^2^ infusion dose died of cardiac arrest; one subject treated with the 14-mg/m^2^ infusion died of bowel perforations; and another subject also treated at the 14-mg/m^2^ dose level died of unknown cause. All 4 AEs leading to death were deemed unlikely related to dinaciclib treatment by the investigator. A total of 6 (13%) subjects reported AEs leading to discontinuation of treatment, but in 4 of the 6 subjects, AEs leading to discontinuation were considered unlikely related to dinaciclib.

### Pharmacodynamics and pharmacokinetics of dinaciclib

**Figure 1:**
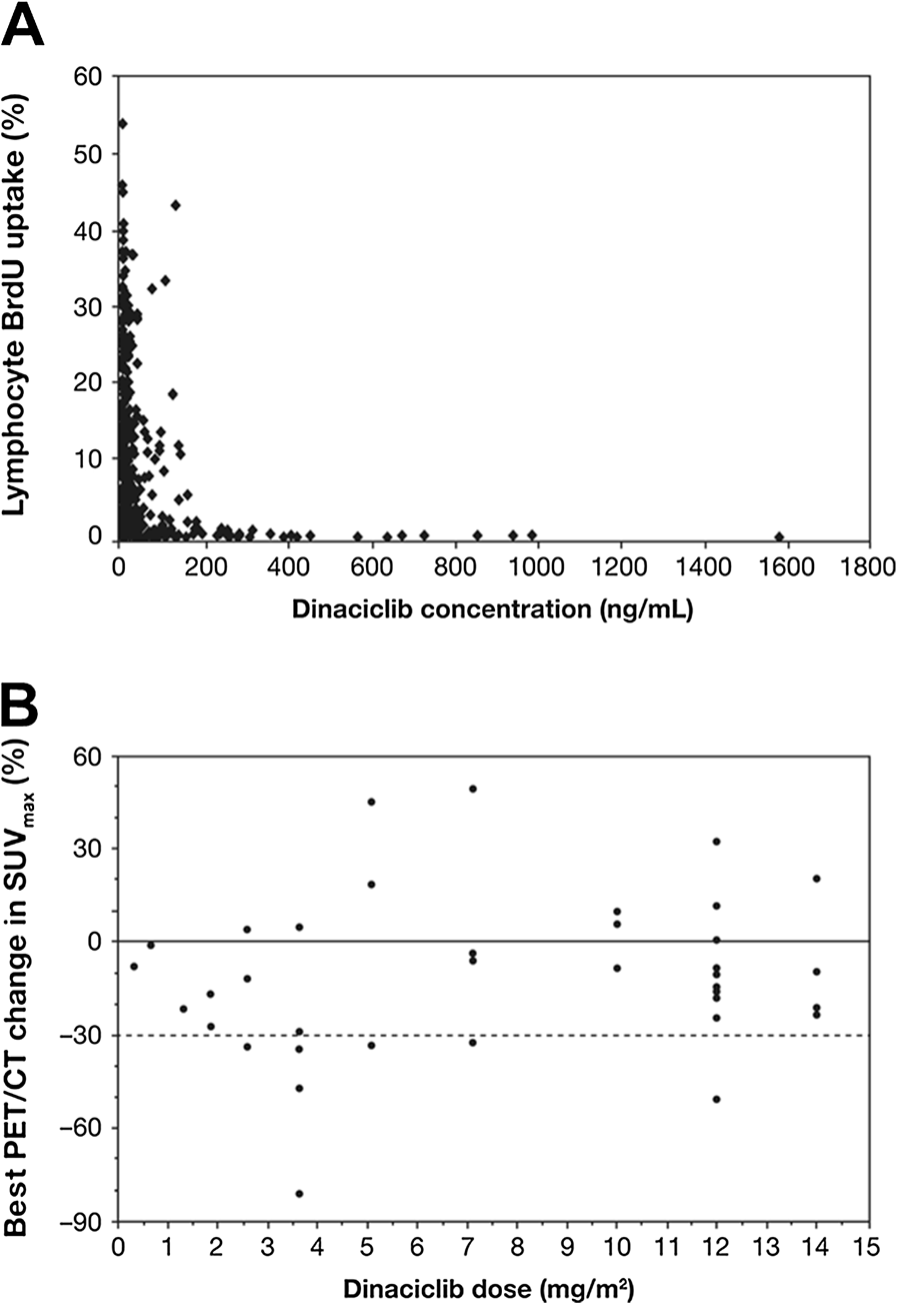
**Pharmacodynamic relationships following treatment with dinaciclib.** Relationship between percentage of bromodeoxyuridine uptake (relative to baseline) and dinaciclib plasma concentration **(A)**, and best percentage change in maximum standard uptake value (SUV_max_) determined by PET/CT scan **(B)** for subjects administered 0.33 to 14.0 mg/m^2^ doses of dinaciclib.

Lymphocyte proliferation (PHA-stimulated%BrdU uptake) data were available from 46 of the 48 treated subjects. Following treatment at the RP2D of 12 mg/m^2^, lymphocyte proliferation was generally inhibited compared with proliferation levels observed pretreatment, although there was some variability (Table 4). The inhibition of ex vivo PHA-stimulated lymphocyte proliferation correlated with the observed plasma concentrations from 46 subjects (Figure 1A). The majority of samples had BrdU incorporation of less than 5% at plasma concentration of 100 ng/mL; BrdU incorporation was completely inhibited at plasma concentration >200 ng/mL. Complete inhibition of BrdU uptake (relative to baseline) was achieved at dinaciclib plasma concentrations greater than 100 ng/mL at about 2 hours after the start of IV infusion with dinaciclib doses of approximately 5.08 mg/m^2^ and greater. Seven subjects were evaluable for BrdU response rate at the RP2D, and all 7 subjects were classified as responders (BrdU response rate: 100%; 2-sided 95% CI: 59%, 100%). Additionally, 10 of the 11 subjects treated with dinaciclib at the RP2D had both pretreatment and cycle 1 day 22 SUV_max_ data, and were therefore evaluable for response by PET/CT analysis. One subject at the RP2D was classified as a PET/CT responder with the best SUV_max_ decrease being greater than 30% (Figure 1B); the PET/CT response rate at the RP2D is 10.0% based on the 10 evaluable subjects (2-sided 95% CI: 0.3%, 44.5%).

**Table 4:** **Differences in mean percent BrdU uptake in ex vivo lymphocyte assays in subjects treated at RP2D of dinaciclib 12 mg/m**^**2**^

**Time points**		**Number of patients**	**Mean percent difference in BrdU uptake (95% CI)**	**Standard error**	**Degrees of freedom**	**t**	***P *value**
Day 1	Predose – 2 hours	7	10.72 (4.69, 16.75)	2.46	6	4.35	0.005
	Predose – 3 hours	7	9.28 (1.44, 17.13)	3.21	6	2.89	0.028
	Predose – 4 hours	7	6.05 (-4.03, 16.12)	4.12	6	1.47	0.192
	Predose – 6 hours	7	10.16 (3.89, 16.43)	2.56	6	3.96	0.007
	Predose – 8 hours	6	8.76 (2.87, 14.65)	2.29	5	3.82	0.012
Day 15	Predose – 2 hours	7	14.82 (6.02, 23.61)	3.59	6	4.12	0.006
	Predose – 3 hours	7	12.95 (2.01, 23.89)	4.47	6	2.90	0.028
	Predose – 4 hours	7	5.47 (-4.17, 15.12)	3.94	6	1.39	0.214
	Predose – 6 hours	6	15.90 (7.69, 24.11)	3.19	5	4.98	0.004
	Predose – 8 hours	5	16.48 (5.63, 27.34)	3.91	4	4.21	0.014

BrdU = bromodeoxyuridine; PHA = phytohemagglutinin; RP2D = recommended phase 2 dose.

Results of paired t-tests showing the difference in mean percent BrdU uptake between predose and postdose ex vivo PHA lymphocyte stimulation assays.

Analysis of subject skin biopsy samples demonstrated pretreatment phospho-Rb staining. Mean IHC scores were calculated before and after treatment for the 11 subjects who were treated at the RP2D of 12 mg/m^2^. Before dinaciclib treatment, these subjects had a mean H score of 18.55; following treatment, the overall H score decreased to 17.64. Therefore, as no subjects demonstrated complete loss of phospho-Rb staining following treatment with dinaciclib, no subjects were deemed to have achieved a response based on phospho-Rb staining, as defined in the study protocol.

**Figure 2:**
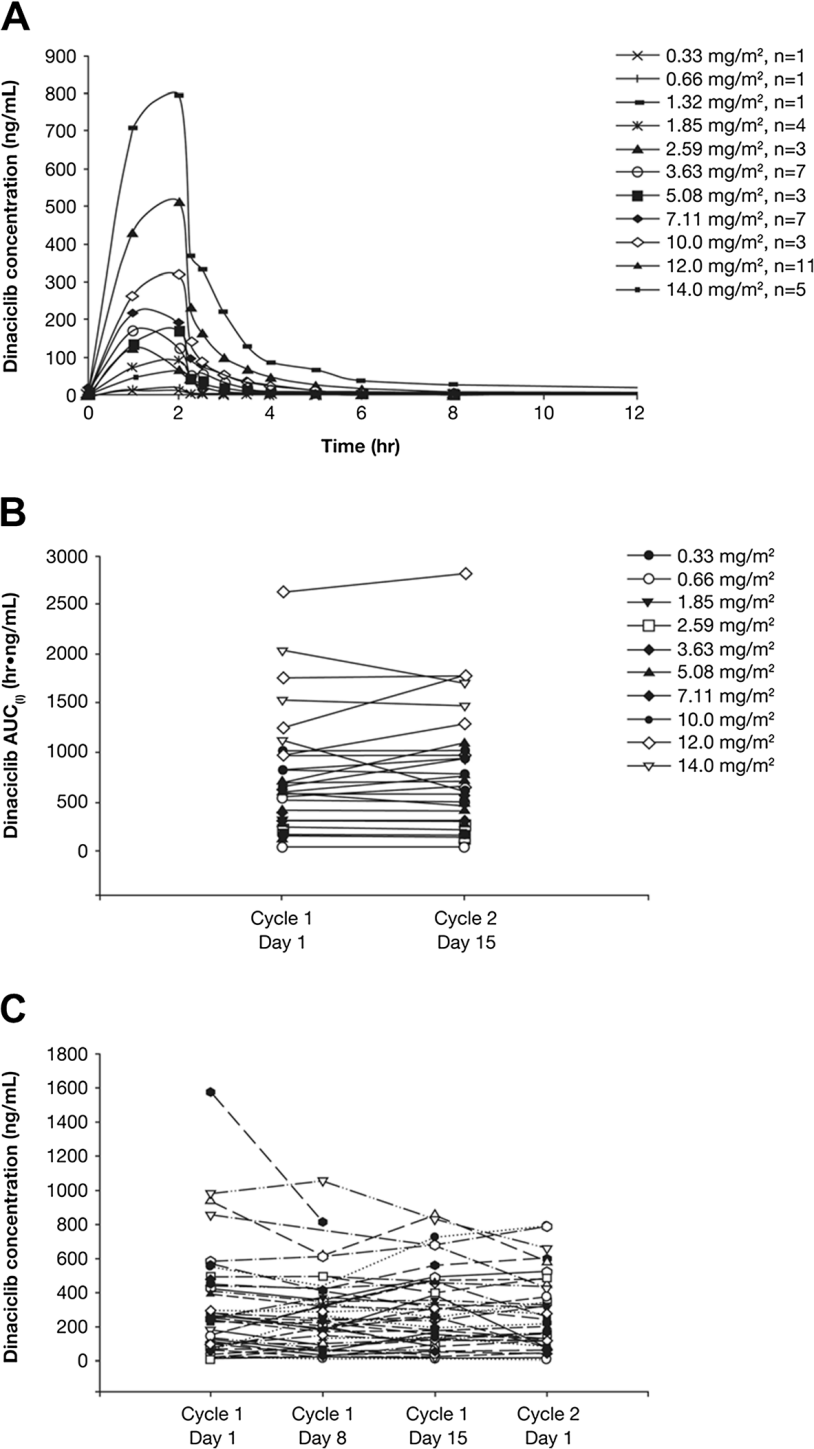
**Pharmacokinetics of dinaciclib following 2**-**hour infusion at different doses.** Mean dinaciclib concentration-time profiles following 2-hour IV infusion on day 1 of cycle 1 **(A)**, comparison of individual AUC_(I)_ on days 1 and 15 of cycle 1 **(B)**, and dinaciclib plasma concentrations at the end of each 2-hour infusion **(C)** (on days 1, 8, and 15 of cycle 1 and on day 1 of cycle 2) for subjects administered dinaciclib at 0.33 mg/m^2^ to 14.0 mg/m^2^. For AUC_(l)_, lines connect day 1 and day 15 values for subjects with data on both days.

Of the 48 treated subjects, 47 subjects were evaluable for the PK analysis; one subject who received IV infusion for less than 1 hour—resulting in less than 3.63 mg/m^2^ dose of dinaciclib on day 1 of cycle 1—and had no concentration-versus-time data on day 15 of cycle 1 was excluded from the analysis. Following 2-hour IV administration of dinaciclib, C_max_ was observed at approximately 2 hours after the initiation of the infusion, and dinaciclib exhibited rapid distribution and elimination phases after the end of an infusion (Figure 2A). Terminal half-life values ranged from 1.5 to 3.6 hours following IV administration of dinaciclib, and CL appeared to be dose independent. Dose-related increases in exposure to dinaciclib were observed as doses increased from 0.33 to 14 mg/m^2^. Exposure to dinaciclib was similar on days 1 and 15 after once-weekly dosing, with a mean AUC_(l)_ ratio (day 15 to day 1) of 1.04 (Figure 2B). Plasma concentrations at the end of each 2-hour infusion (on days 1, 8, and 15 of cycle 1 and on day 1 of cycle 2) were also similar within each subject (Figure 2C). These data suggest that dinaciclib does not accumulate in plasma and pharmacokinetics do not appear to be time dependent over the time course evaluated in this study. Pharmacokinetic parameter means at each dose level, assessed on day 1 and day 15, are available as supplemental information (Additional file 2: Table S2).

### Tumor response

There were no observed complete or partial responses (CR or PR) based on RECIST guidelines in subjects with solid tumors following treatment with dinaciclib. Ten patients achieved stable disease (SD) through at least 4 cycles of treatment with dinaciclib, including 2 subjects with NSCLC and 2 subjects with adenoid cystic carcinoma (Table 5). One subject, with sarcoma, demonstrated prolonged SD through 12 treatment cycles.

**Table 5:** **Patients with stable disease** (**RECIST**) **for at least 4 cycles of treatment with dinaciclib**

**Subject number**	**Diagnosis**	**Dinaciclib dose level (mg/m**^**2**^**)**	**Number of prior treatment regimens**	**Response by RECIST**	**Number of cycles**
2902	NSCLC	0.66	16	SD	6
2910	Pseudomyxoma peritonei	2.59	3	SD	9
2913	Prostate cancer	3.63	2	SD	4
2920	Melanoma	3.63	3	SD	10
2926	Sarcoma	7.11	2	SD	12
2928	Gastrointestinal stromal tumor	7.11	2	SD	10
2931	Esophageal carcinoma	7.11	3	SD	11
2932	Adenoid cyst carcinoma	7.11	0	SD	5
2937	Adenoid cyst carcinoma	14.0	3	SD	8
2945	NSCLC	12.0	4	SD	4

In this study, the CDK inhibitor dinaciclib was administered once weekly for 3 weeks followed by a 1-week recovery period (28-day cycle) and had an acceptable safety and tolerability profile for subjects with solid tumors. The MAD for dinaciclib, administered at a 2-hour IV infusion, was 14 mg/m^2^, and the DLTs experienced at this dose level were orthostatic hypotension and elevated uric acid. Hypotension may be associated with cytokine release syndrome, which has been observed in patients with hematologic malignancies and advanced solid tumors treated with the CDK inhibitor flavopiridol, and has also been identified as a DLT [19,20]. The most frequently reported treatment-related AEs at all dose levels tested were nausea and anemia, and 16 subjects experienced grade 3 or 4 treatment-related AEs. Anemia, neutropenia, and fatigue were the most common AEs related to study drug reported at the RP2D of 12 mg/m^2^. The most frequent SAEs among the 17 subjects who reported experiencing SAEs were deep vein thrombosis, sepsis, and anemia. Adverse events led to the discontinuation of treatment in 6 subjects and 4 subjects died due to AEs that were deemed unrelated to dinaciclib.

Dinaciclib effectively inhibited peripheral blood lymphocyte proliferation, as measured by an ex vivo lymphocyte stimulation assay, demonstrating PD activity when administered at the RP2D (12 mg/m^2^) as a 2-hour IV infusion. One mechanism by which CDK1 and CDK2 may regulate the cell cycle is via phosphorylation of the Rb tumor suppressor family of proteins [4]. In our study, treatment with dinaciclib did not result in substantial decreases in the phosphorylation of the Rb protein in skin biopsies, indicating that no subject had a PD response to dinaciclib treatment based on the protocol-specified criteria that required complete suppression of Rb phosphorylation. It is unlikely that the lack of an observed PD effect using phospho-Rb staining of skin biopsies was due to a limited effect of dinaciclib activity in inhibiting the cell cycle, since dinaciclib treatment inhibited ex vivo lymphocyte proliferation. In preclinical studies, IHC staining of mouse skin biopsies looking at Rb phosphorylation at serine 807 and serine 811 demonstrated strong pretreatment Rb phosphorylation followed by a time-dependent loss of Rb phosphorylation, with a partial loss at 2 hours post treatment and complete loss of Rb phosphorylation at 4 hours post treatment [12,21]. The lack of inhibition of phospho-Rb observed in our trial may be due to the timing of the posttreatment skin biopsy, as the nonclinical data from mice clearly showed a time-dependent effect. Skin biopsies were obtained 4 hours post treatment, on the basis of mouse data, and this may not be the optimal time point in patients.

Our trial enrolled subjects with a variety of solid tumors who were heavily pretreated, as is typical in a phase 1 study population. Early PET/CT scan analysis, as a biomarker for SD, did not show any correlation between tumor metabolic changes and treatment with dinaciclib. Analysis of tumor response using RECIST criteria also showed no objective responses (CR or PR) among the subjects in this study. However, at least 10 subjects achieved prolonged SD for at least 4 cycles of treatment, with one subject demonstrating prolonged SD while receiving treatment for 12 cycles. Therefore, treatment with dinaciclib may have the ability to delay disease progression in some subjects with solid tumors. However, given the small sample size of 48 treated subjects, no clear correlation was observed between day 1/day 15 ex vivo lymphocyte proliferation inhibition (measured by BrdU uptake) and day 22 PET/CT analysis SUV_max_, or between day 22 PET/CT response (SUV_max_ decrease greater than 30%) and the duration of SD. The lack of a correlation could be due to the great heterogeneity among subjects’ baseline characteristics in terms of tumor types, disease stage, and the number of prior chemotherapy regimens. Alternatively, lower concentration and/or shorter duration of drug exposure in the tumors compared with blood may have accounted for the lack of correlation observed in the study.

Several CDK inhibitors have been evaluated in phase 1 clinical trials, but none has demonstrated significant monotherapy activity in solid tumor patients, despite strong preclinical data to support their use. The lack of correlation of antitumor activity observed in vitro and in vivo, in this and other studies, may be affected by dosing schedules and/or drug exposure. The pan-CDK inhibitor flavopiridol was originally studied in 3 phase 1 trials using 2 different schedules. No objective responses were observed in a trial of 55 patients using a 1-hour daily infusion for 5 days, 3 days, or 1 day in a 21-day cycle [22]. However, two trials evaluated flavopiridol with a 72-hour continuous infusion given every 2 weeks, and this schedule resulted in one PR in a patient with renal cancer in a study of 76 patients, and one CR in a patient with gastric cancer in a trial of 38 patients [23,24]. The CDK1, CDK2, and CDK4 inhibitor PHA793887 did not show any objective responses in a first-in-human study in solid tumor patients [25], whereas one PR was observed with the CDK1, CDK2, CDK4, CDK5, and CDK9 inhibitor AT7519 in a patient with metastatic NSCLC [26]. Orally bioavailable CDK inhibitors include the CDK1 and CDK2 inhibitor AZD5438, the CDK1, CDK2, CDK7, and CDK9 inhibitor seliciclib (CYC202; R-roscovitine), and the CDK4 and CDK6 inhibitor PD0332991. Phase 1 trials of these agents report one PR in a patient with testicular cancer among 33 patients treated with PD0332991 [27], and one PR in a patient with hepatocellular carcinoma among 56 patients treated with seliciclib [28]. No responses were observed in 3 phase 1 trials of AZD5438 [29] or in a separate trial of seliciclib [30]. The identification of biomarkers may help to stratify patients into specific groups to determine the predictive response to CDK inhibitors. Preclinical and phase 2 studies have associated elevated expression of Rb protein, luminal ER subtype, and reduced P16 expression with sensitivity to PD033299, a selective inhibitor of CDK4/6 [31-33]. CDK4/CDK6 inhibitors shut down Rb phosphorylation; therefore, responses are precluded in tumor cells that lack Rb. In contrast, to our knowledge, a clear predictive biomarker profile for broad CDK inhibitors has not been identified.

The development of flavopiridol was marked by dose-limiting diarrhea in both 72-hour continuous infusion trials [23,24], and by dose-limiting neutropenia using the daily 1-hour infusion schedule [22]. Several newer CDK inhibitors, such as PD0332991, have also resulted in DLTs of neutropenia [34]. Neutropenia as a DLT has been seen with dinaciclib using higher doses (up to 57 mg/m^2^) on a once-every-21-days dosing schedule [35]. Dose-limiting toxicities with seliciclib, administered orally twice daily for 7 days of a 21-day schedule, were similar to those observed with dinaciclib using the once-weekly dosing schedule, including hypokalemia, hyponatremia, elevated gamma-glutamyl transferase, hyperglycemia, and vasculitic rash (myelosuppression was not observed as a DLT with seliciclib) [30]. The first-in-human trial of PHA793887 administered as a 1-hour infusion on days 1, 8, and 15 in a 4-week cycle resulted in a patient with fatal hepatorenal failure at the third dose level of 44 mg/m^2^ and a patient with grade 4 hepatic failure at the next dose level of 66 mg/m^2^, which led the sponsor to discontinue further development of this agent [25]. Development of AZD5438 was also discontinued due to high variability and unpredictable drug exposure combined with a lack of objective responses [29]. Interestingly AZD5438 was studied first in healthy volunteers with DLT of nausea and vomiting with a single dose of 160 mg [36]; similar AZD5438 exposures (40 mg four times a day) were not tolerated using various continuous daily dosing schedules in the phase 1 trial in advanced solid tumors [29]. It is not clear if the toxicities of AZD5438 and PHA793887 are off-target effects or if they are due to CDK inhibition.

## Conclusions

Several preliminary reports from phase 1 clinical trials have demonstrated enhanced antitumor activity when CDK inhibitors are combined with cytotoxic agents, in patients with both advanced solid tumors [37,38] and estrogen receptor-positive/human epidermal growth factor receptor 2-negative (ER+/HER2–) advanced breast cancer [39]. Initial results from an ongoing phase 2 trial examining the combination of PD-0332991 and letrozole in ER+/HER2– breast cancer patients showed significant improvements in progression-free survival, as well as higher response and clinical benefit rates with the combination compared with letrozole alone [31]. Preclinical studies using tumor cell lines have also shown promising results when CDK inhibitors have been used in combination with other targeted therapies, such as histone deacetylase inhibitors (MS-275 and vorinostat) [40,41] and AKT inhibitors [42]. In early phase clinical trials, dinaciclib has also shown encouraging results as monotherapy in CLL at the RP2D, indicating dinaciclib may also be effective in some hematologic malignancies. Other CDK inhibitors (eg, SNA-032) have not demonstrated similar efficacy in subjects with CLL [43]. These results suggest that dinaciclib combination strategies may be especially promising in solid tumors, and dinaciclib as monotherapy or in combination may also be effective in hematologic malignancies.

## Abbreviations

AE: Adverse event; ALT: Alanine aminotransferase; AST: Aspartate aminotransferase; AUC(I): Area under the plasma concentration-time curve from time zero to infinity; BrdU: Bromodeoxyuridine; CDK: Cyclin-dependent kinase; CI: Confidence interval; CL: Clearance; CLL: Chronic lymphocytic leukemia; Cmax: Maximum observed plasma concentration; CR: Complete response; DLT: Dose-limiting toxicity; ECOG: Eastern Cooperative Oncology Group; ER: Estrogen receptor; FDG-PET/CT: ^18^ F-fluorodeoxyglucose positron emission tomography/computed tomography; HER2: Human epidermal growth factor receptor 2; HIV: Human immunodeficiency virus; IC50: Half maximal inhibitory concentration; IHC: Immunohistochemistry; IV: Intravenous; LC-MS/MS: High performance liquid chromatographic-tandem mass spectrometry; MAD: Maximum administered dose; MedDRA: Medical Dictionary for Regulatory Activities; MRI: Magnetic resonance imaging; NCI CTCAE: National Cancer Institute Common Terminology Criteria for Adverse Events; NSCLC: Non–small cell lung cancer; PD: Pharmacodynamic; PHA: Phytohemagglutinin; PK: Pharmacokinetic; PR: Partial response; R: Accumulation ratio; Rb: Retinoblastoma; RECIST: Response evaluation criteria in solid tumors; RP2D: Recommended phase 2 dose; SAE: Serious adverse event; SD: Stable disease; SUVmax: Standardized uptake value; t½: terminal phase half-life; TEAE: Treatment-emergent adverse event; Tmax: Time of maximum plasma concentration; Vd: Volume of distribution.

## Competing interests

JJN received honoraria as the consulting investigator for the clinical study report and owns shares in Gradalis, Inc.; PK, DZ, and YZ are or were employees of Merck Sharp & Dohme Corp., a subsidiary of Merck & Co., Inc., Whitehouse Station, NJ, when the study was conducted; KAS, Y-MJ, and PS are employees of and own stock in Merck Sharp & Dohme Corp., a subsidiary of Merck & Co., Inc., Whitehouse Station, NJ; S-LY is an employee of Merck Sharp & Dohme Corp., a subsidiary of Merck & Co., Inc., Whitehouse Station, NJ, and owns shares in Gradalis, Inc.; RB was an employee of and owned stock in Merck Sharp & Dohme Corp., a subsidiary of Merck & Co., Inc., Whitehouse Station, NJ, when the study was conducted, and is listed on a pending patent for the study drug dinaciclib.

## Authors’ contributions

JJN collected or assembled data, interpreted the results, wrote sections of the initial draft, provided substantive suggestions for revision or critically reviewed later drafts, and reviewed and confirmed that all relevant competing interests were disclosed. KAS conceived, designed, or planned the study, performed or supervised analyses, interpreted the results, provided substantive suggestions for revision or critically reviewed later drafts, and reviewed and confirmed that all relevant competing interests were disclosed. PK performed or supervised analyses, interpreted the results, wrote sections of the initial draft, and reviewed and confirmed that all relevant competing interests were disclosed. DZ collected or assembled the data, performed or supervised analyses, interpreted the results, wrote sections of the initial draft, and reviewed and confirmed that all relevant competing interests were disclosed. YZ performed or supervised analyses, interpreted the results, and wrote sections of the initial draft. Y-MJ conceived, designed, or planned the study, performed or supervised analyses, interpreted the results, provided substantive suggestions for revision or critically reviewed later drafts, and reviewed and confirmed that all relevant competing interests were disclosed. PS conceived, designed, or planned the study, performed or supervised analyses, interpreted the results, provided substantive suggestions for revision or critically reviewed later drafts, and reviewed and confirmed that all relevant competing interests were disclosed. S-LY conceived, designed, or planned the study, performed or supervised analyses, interpreted the results, provided substantive suggestions for revision or critically reviewed later drafts, and reviewed and confirmed that all relevant competing interests were disclosed. RB conceived, designed, or planned the study, performed or supervised analyses, interpreted the results, wrote sections of the initial draft, provided substantive suggestions for revision or critically reviewed later drafts, and reviewed and confirmed that all relevant competing interests were disclosed. All authors reviewed and approved the final version of the paper.

## Supplementary Material

Additional file 1: Table S1 Mean pharmacokinetic parameters at each dose level. Click here for file

Additional file 2: Table S2 Patients with serious adverse events regardless of causality and according to diagnosis at study entry. Click here for file

## References

[B1] Malumbres M Barbacid M Cell cycle kinases in cancer Curr Opin Genet Dev 2007 17 60 65 10.1016/j.gde.2006.12.008 17208431

[B2] Cicenas J Valius M The CDK inhibitors in cancer research and therapy J Cancer Res Clin Oncol 2011 137 1409 1418 10.1007/s00432-011-1039-4 21877198 PMC11827940

[B3] Malumbres M Barbacid M To cycle or not to cycle: a critical decision in cancer Nat Rev Cancer 2001 1 222 231 10.1038/35106065 11902577

[B4] Shapiro GI Cyclin-dependent kinase pathways as targets for cancer treatment J Clin Oncol 2006 24 1770 1783 10.1200/JCO.2005.03.7689 16603719

[B5] Abdullah C Wang X Becker D Expression analysis and molecular targeting of cyclin-dependent kinases in advanced melanoma Cell Cycle 2011 10 977 988 10.4161/cc.10.6.15079 21358262 PMC3100877

[B6] McMillin DW Delmore J Negri J Buon L Jacobs HM Laubach J Jakubikova J Ooi M Hayden P Schlossman R Munshi NC Lengauer C Richardson PG Anderson KC Mitsiades CS Molecular and cellular effects of multi-targeted cyclin-dependent kinase inhibition in myeloma: biological and clinical implications Br J Haematol 2011 152 420 432 10.1111/j.1365-2141.2010.08427.x 21223249 PMC4042406

[B7] Quereda V Malumbres M Cell cycle control of pituitary development and disease J Mol Endocrinol 2009 42 75 86 18987159 10.1677/JME-08-0146

[B8] Hahntow IN Schneller F Oelsner M Weick K Ringshausen I Fend F Peschel C Decker T Cyclin-dependent kinase inhibitor Roscovitine induces apoptosis in chronic lymphocytic leukemia cells Leukemia 2004 18 747 755 10.1038/sj.leu.2403295 14973497

[B9] Johnson N Shapiro GI Cyclin-dependent kinases (cdks) and the DNA damage response: rationale for cdk inhibitor-chemotherapy combinations as an anticancer strategy for solid tumors Expert Opin Ther Targets 2010 14 1199 1212 10.1517/14728222.2010.525221 20932174 PMC3957489

[B10] Sutherland RL Musgrove EA CDK inhibitors as potential breast cancer therapeutics: new evidence for enhanced efficacy in ER + disease Breast Cancer Res 2009 11 112 10.1186/bcr2454 20067604 PMC2815549

[B11] Wesierska-Gadek J Maurer M Promotion of apoptosis in cancer cells by selective purine-derived pharmacological CDK inhibitors: one outcome, many mechanisms Curr Pharm Des 2011 17 256 271 10.2174/138161211795049714 21348827

[B12] Parry D Guzi T Shanahan F Davis N Prabhavalkar D Wiswell D Seghezzi W Paruch K Dwyer MP Doll R Nomeir A Windsor W Fischmann T Wang Y Oft M Chen T Kirschmeier P Lees EM Dinaciclib (SCH 727965), a novel and potent cyclin-dependent kinase inhibitor Mol Cancer Ther 2010 9 2344 2353 10.1158/1535-7163.MCT-10-0324 20663931

[B13] Paruch K Dwyer MP Alvarez C Brown C Chan T-Y Doll RJ Keertikar K Knutson C McKittrick B Rivera J Rossman R Tucker G Fischmann T Hruza A Madison V Nomeir AA Wang Y Kirschmeier P Lees E Parry D Sgambellone N Seghezzi W Schultz L Shanahan F Wiswell D Xu X Zhou Q James RA Paradkar VM Park H Discovery of dinaciclib (SCH 727965): a potent and selective inhibitor of cyclin-dependent kinases ACS Med Chem Lett 2010 1 204 208 10.1021/ml100051d 24900195 PMC4007794

[B14] Fu W Ma L Chu B Wang X Bui MM Gemmer J Altiok S Pledger WJ The cyclin-dependent kinase inhibitor SCH 727965 (dinacliclib) induces the apoptosis of osteosarcoma cells Mol Cancer Ther 2011 10 1018 1027 10.1158/1535-7163.MCT-11-0167 21490307 PMC4727401

[B15] Feldmann G Mishra A Bisht S Karikari C Garrido-Laguna I Rasheed Z Ottenhof NA Dadon T Alvarez H Fendrich V Rajeshkumar NV Matsui W Brossart P Hidalgo M Bannerji R Maitra A Nelkin BD Cyclin-dependent kinase inhibitor dinaciclib (SCH727965) inhibits pancreatic cancer growth and progression in murine xenograft models Cancer Biol Ther 2011 12 598 609 10.4161/cbt.12.7.16475 21768779 PMC3218385

[B16] Simon R Freidlin B Rubinstein L Arbuck SG Collins J Christian MC Accelerated titration designs for phase I clinical trials in oncology J Natl Cancer Inst 1997 89 1138 1147 10.1093/jnci/89.15.1138 9262252

[B17] Zhang D Mita M Shapiro GI Poon J Small K Tzontcheva A Kantesaria B Zhu Y Bannerji R Statkevich P Effect of aprepitant on the pharmacokinetics of the cyclin-dependent kinase inhibitor dinaciclib in patients with advanced malignancies Cancer Chemother Pharmacol 2012 70 891 898 10.1007/s00280-012-1967-y 23053255

[B18] Eisenhauer EA Therasse P Bogaerts J Schwartz LH Sargent D Ford R Dancey J Arbuck S Gwyther S Mooney M Rubinstein L Shankar L Dodd L Kaplan R Lacombe D Verweij J New response evaluation criteria in solid tumours: revised RECIST guideline (version 1.1) Eur J Cancer 2009 45 228 247 10.1016/j.ejca.2008.10.026 19097774

[B19] Phelps MA Lin TS Johnson AJ Hurh E Rozewski DM Farley KL Wu D Blum KA Fischer B Mitchell SM Moran ME Brooker-McEldowney M Heerema NA Jarjoura D Schaaf LJ Byrd JC Grever MR Dalton JT Clinical response and pharmacokinetics from a phase 1 study of an active dosing schedule of flavopiridol in relapsed chronic lymphocytic leukemia Blood 2009 113 2637 2645 10.1182/blood-2008-07-168583 18981292 PMC2661854

[B20] Ramaswamy B Phelps MA Baiocchi R Bekaii-Saab T Ni W Lai JP Wolfson A Lustberg ME Wei L Wilkins D Campbell A Arbogast D Doyle A Byrd JC Grever MR Shah MH A dose-finding, pharmacokinetic and pharmacodynamic study of a novel schedule of flavopiridol in patients with advanced solid tumors Invest New Drugs 2012 30 629 638 10.1007/s10637-010-9563-7 20938713 PMC3486515

[B21] Berenson JR Hillner BE Kyle RA Anderson K Lipton A Yee GC Biermann JS American Society of Clinical Oncology clinical practice guidelines: the role of bisphosphonates in multiple myeloma J Clin Oncol 2002 20 3719 3736 10.1200/JCO.2002.06.037 12202673

[B22] Tan AR Headlee D Messmann R Sausville EA Arbuck SG Murgo AJ Melillo G Zhai S Figg WD Swain SM Senderowicz AM Phase I clinical and pharmacokinetic study of flavopiridol administered as a daily 1-hour infusion in patients with advanced neoplasms J Clin Oncol 2002 20 4074 4082 10.1200/JCO.2002.01.043 12351605

[B23] Senderowicz AM Headlee D Stinson SF Lush RM Kalil N Villalba L Hill K Steinberg SM Figg WD Tompkins A Arbuck SG Sausville EA Phase I trial of continuous infusion flavopiridol, a novel cyclin-dependent kinase inhibitor, in patients with refractory neoplasms J Clin Oncol 1998 16 2986 2999 9738567 10.1200/JCO.1998.16.9.2986

[B24] Thomas JP Tutsch KD Cleary JF Bailey HH Arzoomanian R Alberti D Simon K Feierabend C Binger K Marnocha R Dresen A Wilding G Phase I clinical and pharmacokinetic trial of the cyclin-dependent kinase inhibitor flavopiridol Cancer Chemother Pharmacol 2002 50 465 472 10.1007/s00280-002-0527-2 12451473

[B25] Massard C Soria JC Anthoney DA Proctor A Scaburri A Pacciarini MA Laffranchi B Pellizzoni C Kroemer G Armand JP Balheda R Twelves CJ A first in man, phase I dose-escalation study of PHA-793887, an inhibitor of multiple cyclin-dependent kinases (CDK2, 1 and 4) reveals unexpected hepatotoxicity in patients with solid tumors Cell Cycle 2011 10 963 970 10.4161/cc.10.6.15075 21368575

[B26] Mahadevan D Plummer R Squires MS Rensvold D Kurtin S Pretzinger C Dragovich T Adams J Lock V Smith DM Von Hoff D Calvert H A phase I pharmacokinetic and pharmacodynamic study of AT7519, a cyclin-dependent kinase inhibitor in patients with refractory solid tumors Ann Oncol 2011 22 2137 2143 10.1093/annonc/mdq734 21325451

[B27] Schwartz GK LoRusso PM Dickson MA Randolph SS Shaik MN Wilner KD Courtney R O’Dwyer PJ Phase I study of PD 0332991, a cyclin-dependent kinase inhibitor, administered in 3-week cycles (Schedule 2/1) Br J Cancer 2011 104 1862 1868 10.1038/bjc.2011.177 21610706 PMC3111206

[B28] Le Tourneau C Faivre S Laurence V Delbaldo C Vera K Girre V Chiao J Armour S Frame S Green SR Gianella-Borradori A Dieras V Raymond E Phase I evaluation of seliciclib (R-roscovitine), a novel oral cyclin-dependent kinase inhibitor, in patients with advanced malignancies Eur J Cancer 2010 46 3243 3250 10.1016/j.ejca.2010.08.001 20822897

[B29] Boss DS Schwartz GK Middleton MR Amakye DD Swaisland H Midgley RS Ranson M Danson S Calvert H Plummer R Morris C Carvajal RD Chirieac LR Schellens JH Shapiro GI Safety, tolerability, pharmacokinetics and pharmacodynamics of the oral cyclin-dependent kinase inhibitor AZD5438 when administered at intermittent and continuous dosing schedules in patients with advanced solid tumours Ann Oncol 2010 21 884 894 10.1093/annonc/mdp377 19825886 PMC2844945

[B30] Benson C White J De Bono J O’Donnell A Raynaud F Cruickshank C McGrath H Walton M Workman P Kaye S Cassidy J Gianella-Borradori A Judson I Twelves C A phase I trial of the selective oral cyclin-dependent kinase inhibitor seliciclib (CYC202; R-Roscovitine), administered twice daily for 7 days every 21 days Br J Cancer 2007 96 29 37 10.1038/sj.bjc.6603509 17179992 PMC2360206

[B31] Finn RS Crown JP Lang I Boer K Bondarenko IM Kulyk SO Ettl J Patel R Pinter T Schmidt M Shparyk Y Thummala AR Voytko NL Breazna A Kim ST Randolph S Slamon DJ Results of a randomized phase 2 study of PD 0332991, a cyclindependent kinase (CDK) 4/6 inhibitor, in combination with letrozole vs letrozole alone for first-line treatment of ER+/HER2- advanced breast cancer (BC) Cancer Res 2012 72 S1 S6 Abstract

[B32] Finn RS Dering J Conklin D Kalous O Cohen DJ Desai AJ Ginther C Atefi M Chen I Fowst C Los G Slamon DJ PD 0332991, a selective cyclin D kinase 4/6 inhibitor, preferentially inhibits proliferation of luminal estrogen receptor-positive human breast cancer cell lines in vitro Breast Cancer Res 2009 11 R77 10.1186/bcr2419 19874578 PMC2790859

[B33] Konecny GE Winterhoff B Kolarova T Qi J Manivong K Dering J Yang G Chalukya M Wang HJ Anderson L Kalli KR Finn RS Ginther C Jones S Velculescu VE Riehle D Cliby WA Randolph S Koehler M Hartmann LC Slamon DJ Expression of p16 and retinoblastoma determines response to CDK4/6 inhibition in ovarian cancer Clin Cancer Res 2011 17 1591 1602 10.1158/1078-0432.CCR-10-2307 21278246 PMC4598646

[B34] Schwartz GK Shah MA Targeting the cell cycle: a new approach to cancer therapy J Clin Oncol 2005 23 9408 9421 10.1200/JCO.2005.01.5594 16361640

[B35] Mita MM Mita AC Moseley J Poon J Small KA Jou Y Kirschmeier P Zhang D Statkevich P Sankhala KK Sarantopoulos J Cleary JM Chirieac LR Rodig S Bannerji R Shapiro G A phase I study of the CDK inhibitor dinaciclib (SCH 727965) administered every 3 weeks in patients (pts) with advanced malignancies: final results J Clin Oncol 2011 29 Abstract 3080

[B36] Camidge DR Smethurst D Growcott J Barrass NC Foster JR Febbraro S Swaisland H Hughes A A first-in-man phase I tolerability and pharmacokinetic study of the cyclin-dependent kinase-inhibitor AZD5438 in healthy male volunteers Cancer Chemother Pharmacol 2007 60 391 398 10.1007/s00280-006-0371-x 17115157

[B37] Bahleda R Spreafico A Soria J Moldovan C Belli C Fiorentini F Scaburri A Pacciarini MA Laffranchi B Reni M Phase I study of the oral CDK-TRKA inhibitor PHA-848125 in combination with gemcitabine in advanced solid tumors J Clin Oncol 2010 28 Abstract 3063

[B38] Dickson MA Shah MA Rathkopf D Tse A Carvajal RD Wu N Lefkowitz RA Gonen M Cane LM Dials HJ Schwartz GK A phase I clinical trial of FOLFIRI in combination with the pan-cyclin-dependent kinase (CDK) inhibitor flavopiridol Cancer Chemother Pharmacol 2010 66 1113 1121 10.1007/s00280-010-1269-1 20953860 PMC2957673

[B39] Slamon DJ Hurvitz SA Applebaum S Glaspy JA Allison MK DiCarlo BA Courtney RD Kim ST Randolph SR Finn S Phase I study of PD 0332991, cyclin-D kinase (CDK) 4/6 inhibitor in combination with letrozole for first-line treatment of patients with ER-positive, HER2-negative breast cancer J Clin Oncol 2010 28 Abstract 3060

[B40] Gahr S Peter G Wissniowski TT Hahn EG Herold C Ocker M The histone-deacetylase inhibitor MS-275 and the CDK-inhibitor CYC-202 promote anti-tumor effects in hepatoma cell lines Oncol Rep 2008 20 1249 1256 18949429

[B41] Huang JM Sheard MA Ji L Sposto R Keshelava N Combination of vorinostat and flavopiridol is selectively cytotoxic to multidrug-resistant neuroblastoma cell lines with mutant TP53 Mol Cancer Ther 2010 9 3289 3301 10.1158/1535-7163.MCT-10-0562 21159612 PMC8312524

[B42] Mohapatra S Chu B Zhao X Djeu J Cheng JQ Pledger WJ Apoptosis of metastatic prostate cancer cells by a combination of cyclin-dependent kinase and AKT inhibitors Int J Biochem Cell Biol 2009 41 595 602 10.1016/j.biocel.2008.07.013 18708158

[B43] Tong WG Chen R Plunkett W Siegel D Sinha R Harvey RD Badros AZ Popplewell L Coutre S Fox JA Mahadocon K Chen T Kegley P Hoch U Wierda WG Phase I and pharmacologic study of SNS-032, a potent and selective Cdk2, 7, and 9 inhibitor, in patients with advanced chronic lymphocytic leukemia and multiple myeloma J Clin Oncol 2010 28 3015 3022 10.1200/JCO.2009.26.1347 20479412 PMC4979218

