# Supplementary material for: A first-in-human, phase 1, dose-escalation study of dinaciclib, a novel cyclin-dependent kinase inhibitor, administered weekly in subjects with advanced malignancies
Source: J Transl Med. 2013 Oct 16;11:259. doi: 10.1186/1479-5876-11-259 (PMC3853718; doi:10.1186/1479-5876-11-259)
Supplement: Additional file 1: Table S1 — Mean pharmacokinetic parameters at each dose level. [file 1479-5876-11-259-S1.doc]

**SUPPLEMENTAL MATERIAL**

**Additional file 1:Table S1:** Mean pharmacokinetic parameters at each dose level

AUC=area under the plasma concentration-time curve; AUC(0-24)=AUC from time 0 to 24 hours; AUC(I)=AUC extrapolated to infinity; AUC(tf)=AUC to the final measurable sampling time; CL=clearance; Cmax=maximum observed plasma concentration; CV=coefficient of variation; NA = not available; R = accumulation ratio; t½ = terminal phase half-life; Tmax = time of maximum plasma concentration; Vd = volume of distribution.

| **Day** | **Dose**  **(mg/m^2^)** | **N** | **AUC(0-24 hr) (hr*ng/mL)** | | **AUC(I) (hr*ng/mL)** | | **AUC(tf) (hr*ng/mL)** | | **R** | | **CL (L/hr/m^2^)** | | **Cmax**  **(ng/mL)** | | **Tmax**  **(hr)** | **Vd**  **(L/m^2^)** | | **t**½  **(hr)** | |
| --- | --- | --- | --- | --- | --- | --- | --- | --- | --- | --- | --- | --- | --- | --- | --- | --- | --- | --- | --- |
|  |  |  | **Mean** | **CV%** | **Mean** | **CV%** | **Mean** | **CV%** | **Mean** | **CV%** | **Mean** | **CV%** | **Mean** | **CV%** | **Median** | **Mean** | **CV%** | **Mean** | **CV%** |
| **1** | 0.33 | 1 | 27.8 | NA | 26.7 | NA | 26.1 | NA | NA | NA | 12.4 | NA | 11.1 | NA | 1 | 35.9 | NA | 2.01 | NA |
|  | 0.66 | 1 | 36.8 | NA | 35.7 | NA | 35.3 | NA | NA | NA | 18.5 | NA | 18.6 | NA | 2 | 39.4 | NA | 1.48 | NA |
|  | 1.32 | 1 | 117 | NA | NA^a^ | NA | 112 | NA | NA | NA | NA^a^ | NA | 63.8 | NA | 2 | NA^a^ | NA | NA^a^ | NA |
|  | 1.85 | 4 | 209 | 32 | 222^b^ | 34 | 207 | 34 | NA | NA | 9.16^b^ | 40 | 98.1° | 26 | 2^c^ | 30.2^b^ | 11 | 2.57^b^ | 41 |
|  | 2.59 | 3 | 805 | 130 | 214^d^ | NA | 809 | 130 | NA | NA | 12.8^d^ | NA | 100^e^ | NA | 1^e^ | 61.4^d^ | NA^1^ | 3.28^d^ | NA |
|  | 3.63^g^ | 7 | 464 | 47 | 458^h^ | 52 | 465 | 47 | NA | NA | 11.2^h^ | 71 | 210^i^ | 39 | 1^i^ | 40.2^h^ | 41 | 2.9^h^ | 26 |
|  | 5.08 | 3 | 327 | 21 | 327 | 21 | 327 | 21 | NA | NA | 15.9 | 19 | 173 | 39 | 2 | 72.7 | 13 | 3.19 | 7.3 |
|  | 7.11 | 7 | 542 | 26 | 543 | 26 | 542 | 26 | NA | NA | 14.1 | 32 | 250^j^ | 36 | 2^j^ | 61.7 | 33 | 3.04 | 7.5 |
|  | 10 | 3 | 694 | 41 | 687 | 42 | 681 | 43 | NA | NA | 16.1 | 34 | 319 | 33 | 2 | 68.5 | 35 | 3.02 | 21 |
|  | 12 | 11 | 1150 | 49 | 1150 | 50 | 1150 | 50 | NA | NA | 12.1 | 34 | 525 | 45 | 2 | 55.4 | 31 | 3.28 | 21 |
|  | 14 | 5 | 2020 | 71 | 2020 | 71 | 2020 | 71 | NA | NA | 9.24 | 49 | 821 | 55 | 1 | 39.8 | 45 | 3.09 | 11 |
| **15** | 0.33 | 1 | 42.8 | NA | 43 | NA | 42.8 | NA | 1.54 | NA | 7.67 | NA | NA^k^ | NA | NA^k^ | 51 | NA | 4.61 | NA |
|  | 0.66 | 1 | 28.4 | NA | 27.6 | NA | 27.2 | NA | 0.772 | NA | 23.9 | NA | 13.2 | NA | 1 | 57.6 | NA | 1.67 | NA |
|  | 1.32 | 1 | 134 | NA | 134 | NA | 134 | NA | 1.15 | NA | 9.83 | NA | 69.5 | NA | 1 | 48 | NA | 3.38 | NA |
|  | 1.85 | 3 | 216 | 41 | 216 | 41 | 216 | 42 | 0.953 | 12 | 10 | 54 | 108^1^ | NA | 2^l^ | 33.1 | 3.7 | 2.66 | 39 |
|  | 2.59 | 3 | 735 | 130 | 187^m^ | NA | 739 | 130 | 0.844 | NA^n^ | 15.6^m^ | NA | 104 | 66 | 1 | 80.7^m^ | NA | 3.6^m^ | NA |
|  | 3.63 | 6 | 405 | 57 | 431° | 64 | 401 | 57 | 0.841 | 24 | 12.5° | 80 | 163 | 59 | 1 | 71.6° | 99 | 3.59° | 21 |
|  | 5.08 | 2 | 354 | NA | 354 | NA | 354 | NA | 1.05 | NA^p^ | 14.6 | NA | 147 | NA | 2 | 66.2 | NA | 3.17 | NA |
|  | 7.11 | 7 | 727 | 40 | 686^q^ | 43 | 710 | 39 | 1.36 | 42 | 12.4^q^ | 49 | 306 | 32 | 1 | 49.9^q^ | 64 | 2.71^q^ | 22 |
|  | 10 | 3 | 715 | 39 | 722 | 38 | 704 | 40 | 1.04 | 9 | 15.2 | 35 | 321 | 37 | 2 | 90.6 | 41 | 4.14 | 28 |
|  | 12 | 10 | 1260 | 52 | 1270 | 53 | 1270 | 53 | 1.09 | 15 | 11.5 | 40 | 526 | 40 | 2 | 49.3 | 31 | 3.16 | 21 |
|  | 14 | 4 | 1180 | 42 | 1180 | 42 | 1180 | 42 | 0.832 | 25 | 13.9 | 50 | 568 | 36 | 2 | 70.2 | 58 | 3.43 | 11 |

^a^n=0: AUC(I), CL, t½, and Vd were not determinable for one subject on Cycle 1 Day 1.

^b^n=3: AUC(I), CL, t½, and Vd were not determinable for one subject on Cycle 1 Day 1.

^c^n=3: Tmax and Cmax: end-of-infusion (2-hour) sample was collected after the end of the infusion for one subject on Cycle 1 Day 1.

^d^n=2: AUC(I), CL, t½, and Vd were not determinable for one subject on Cycle 1 Day 1.

^e^n=1: Tmax and Cmax: end-of-infusion (2-hour) sample was collected after the end of the infusion for one subject, and end-of-infusion (2-hour) sample was not collected for one subject on Cycle 1 Day 1.

^f^One subject does not have Day 1 data.

^g^One subject not included in descriptive statistics. This subject received a <1-hour infusion and <3.63-mg/m2 dose on Cycle 1 Day 1.

^h^n=6: AUC(I), CL, t½, and Vd were not determinable for one subject on Cycle 1 Day 1.

^i^n=6: Tmax and Cmax: end-of-infusion 2-hour sample was not collected for one subject on Cycle 1 Day 1.

^j^n=6: Tmax and Cmax: end-of-infusion (2-hour) sample was collected after the end of the infusion for one subject on Cycle 1 Day 1.

^k^n=0: Tmax and Cmax: end-of-infusion (2-hour) sample was collected after the end of the infusion for one subject on Cycle 1 Day 15.

^l^n=2: Tmax and Cmax: end-of-infusion (2-hour) sample was collected after the end of the infusion for one subject on Cycle 1 Day 15.

^m^n=2: AUC(I), CL, t½, and Vd were not determinable for one subject on Cycle 1 Day 15.

^n^One subject does not have Day 15 data.

^o^n=3: AUC(I), CL, t½, and Vd were not determinable for three subjects on Cycle 1 Day 15.

^p^One subject does not have Day 15 data.

^q^n=6: AUC(I), CL, t½, and Vd were not determinable for one subject on Cycle 1 Day 15.
