# Supplementary material for: A first-in-human, phase 1, dose-escalation study of dinaciclib, a novel cyclin-dependent kinase inhibitor, administered weekly in subjects with advanced malignancies
Source: J Transl Med. 2013 Oct 16;11:259. doi: 10.1186/1479-5876-11-259 (PMC3853718; doi:10.1186/1479-5876-11-259)
Supplement: Additional file 2: Table S2 — Patients with serious adverse events regardless of causality and according to diagnosis at study entry. [file 1479-5876-11-259-S2.doc]

**Additional file 2:Table S2.** Patients with serious adverse events regardless of causality and according to diagnosis at study entry

| **Subject number** | **Diagnosis** | **Dinaciclib dose level (mg/m^2^)** | **Serious adverse events** |
| --- | --- | --- | --- |
| 2901 | Breast carcinoma | 0.33 | Anemia, confusion, lethargy |
| 2914 | Ovarian epithelial carcinoma | 3.63 | Constipation, nausea, abdominal pain, vomiting |
| 2915 | Adenocarcinoma | 3.63 | Hypocalcemia, hypokalemia, hypophosphatemia, coagulation time prolonged |
| 2919 | Colon cancer | 3.63 | Back pain, chest pain, dyspnea |
| 2920 | Melanoma | 3.63 | Cholelithiasis, liver disorder |
| 2922 | Ovarian epithelial cancer | 3.63 | Intestinal ischemia, pericardial effusion |
| 2926 | Pelvic sarcoma | 7.11 | Deep vein thrombosis, anemia |
| 2927 | Gastric adenocarcinoma | 7.11 | Vomiting, aspiration |
| 2930 | SCLC | 7.11 | Cardiac arrest |
| 2937 | Adenoid cyst carcinoma | 14 | Pneumothorax, cardiac tamponade, pneumoperitoneum, intestinal perforation, peritonitis, sepsis |
| 2945 | NSCLC | 12 | Cerebrovascular accident |
| 2928 | Gastrointestinal stromal tumor | 7.11 | Gastric ulcer, hematemesis, asthenia, sepsis, dyspnea, mental status changes, overdose |
| 2931 | Esophageal carcinoma | 7.11 | Esophageal obstruction, pyrexia, hemorrhagic anemia |
| 2940 | Breast carcinoma | 14 | Death |
| 2947 | Colorectal cancer | 12 | Pelvic abscess, septic shock, deep vein thrombosis |
| 2950 | Colon cancer | 12 | Deep vein thrombosis, bradycardia, respiratory failure, syncope, tumor lysis syndrome |
| 2951 | Ovarian metastatic cancer | 12 | Anemia, neutropenic sepsis, sepsis, gastrointestinal hemorrhage, diarrhea, bacterial infection, neutropenia, thrombocytopenia |
